# Resolutions of the American Society of Dental Surgeons, in Relation to the Use of Amalgams for Filling Teeth

**Published:** 1845-12

**Authors:** 


### *Resolutions of the* American Society of Dental Surgeons *in relation to the use of Amalgams for Filling Teeth.*

By resolutions passed by the American Society of Dental Surgeons, at its Annual Meeting, held in the city of New York, August, 1845, it was made our duty, as Recording Secretary, to forward to every member of said Society, a certificate, demanding his views upon the use of Amalgams for filling teeth. These resolutions made it binding on the faith of every member receiving such certificate, to sign, and send the same to us, within a certain specified time. This duty we have faithfully performed, and have received from a majority of the members these certificates, duly signed, in accordance with said resolutions of the Society. But, from several members, these letters have met a different response, in far fewer cases than was expected by the Society, when the measure was adopted. How many have silently refused to comply with the request of the Society, by simply non-compliance, we cannot say, there being good reason for believing that many have never received the circular, and the failure, hence, to comply with the resolutions, being no fault of their own. In consideration of this fact, we take this occasion to request all who have not received a copy of the circular, or who were absent till the expiration of the sixty days given by the Society, to write us immediately, giving us a statement of the fact. Although the resolutions are such as to make no exception, yet, the Society may, by future action, reinstate such as are willing to comply, and actually do so, before the next annual meeting, without the formality of them again joining, as prescribed by the constitution and by-laws, as new members. This suggestion applies, of course, only to those who were prevented, during the limited time, from compliance with the resolutions, by circumstances which they could not control. But, as we have already intimated, there are some who have *refused* to sign the certificate, for reasons which are deserving of notice. This is the more demanded, since the chief objections alluded to are sustained and renewed by the "Virginia Society of Surgeon Dentists," in resolutions passed at their last annual meeting. As these objections are not based upon the merits or demerits of the *article* in question, the objections, with only one exception, *professing* to condemn all amalgams, for dental purposes, their discussion becomes one rather belonging to the department of jurisprudence than to dentistry—and we need not say that this is a field of discussion we have never cultivated, and one which we shall not now pretend to enter, only in the way of noticing some plain principles, concerning which, "the wayfaring man need not err." As the *use* of amalgams for dental purposes, is pronounced to be malpractice, not only by those, with one exception, who have refused to sign the pledge, and by the Virginia Society, but by almost every dentist having any claim to respectability throughout the land, but a single question is left for discussion, viz. Had the American Society a RIGHT to adopt her own measures to free herself of those members whose *opinions* and *practice* is averse to her known rules and regulations.

After the most careful examination we have been enabled to give this question, we are prepared to take the position, that the American Society not only *had a most undoubted right* to pass these resolutions, but that it was the only policy which could be adopted, with the hope to bring about the result aimed at. But before proceeding to offer any considerations in support of this position, we shall give, more at length, the views of some of those who entertain an opposite opinion, by quoting from their letters, in in answer to the offensive circular of the Society.

The following is from a Western brother:

"To A. WESTCOTT,

*Recording Secretary of the American Society of Dental Surgeons.*

MY DEAR SIR; The printed resolutions and blank protest, of the American Society of Dental Surgeons, was received, in due time. In reply, I have only to say, that *my* professional attainments and character, occupy higher ground than to be compelled to forfeit my self-respect, by submitting to the requirements of resolutions, whose *tone* is *threatening*. The society has transcended its powers, has violated the compact which ushered it into existence, by enacting resolutions, which are arbitrary, unjust, and unconstitutional, and I believe unless they are abandoned, *the fate of the society is sealed*. *I* do not use any amalgam, or cement, whatever, and have, invariably, when consulted by my professional brethren in the west, recommended the following substitutes, viz. gold, and platina. Gold, however, has advantages over all other substances Tin and lead are admissible. Tin possesses advantages over lead. The demand for lead is less frequent. In my own practice gold has always been preferred, and *extensively* used. Platina, tin, and lead, have also been used, but only to a limited extent, particularly the latter. When the society recommended to the profession, to abandon the use of amalgams, and cements, for the purpose of filling teeth, it did not travel out of the bounds of its duty—this recommendation was right, and proper, and every honorable, and high-minded practitioner of dental surgery, whether a member of the society or not, I venture to aver, was to be found on the side of the society, and opposed to the *indiscriminate* use of amalgams, cements, &c. &c., but when the society declare the use of these objectionable materials, to be *empiricism*, it goes one step too far. I saw that declaration with much regret, and when I read the resolutions, and protest of the last annual meeting, I confess, it was with feelings of pain and mortification. In conclusion, I will say, that my membership of the society does not, I humbly trust, rest upon such a doubtful foundation, as to subject me to the liability of expulsion, unless I sign a certificate, and make a pledge. When a member is supposed to have violated the constitution, or by-laws of the society, *he should be arraigned* under their provisions, and if found guilty, expelled. I will now rest the matter for the present, and await the issue."

I shall not stop here to comment upon the above letter, other than to observe, that all of these loud complaints are made by one, who takes some pains to declare, in the very same breath, that *he* never uses mineral paste, nor does he countenance its use; thus tacitly complying with every requisition of the society, against whose action upon this matter, he so loudly declaims.

The following is from a correspondent in reply to the circular, who considers the *article* in question, not quite so bad, as has been represented, or that it *may be* judiciously used. It will be perceived, however, that the *tone* of his objection, to the *circular*, is precisely the same as in the one just given. I shall only quote so much of this letter, (it being long,) as will exhibit his precise objection to the measure. He says, "the society have certainly transcended their powers, and have taken an injudicious course, to put down the practice, and I think the article of amalgam, would not have obtained half so much, had there not "so much fuss" been made about it. I defy any one to find a precedent for this course. Was it ever heard, that a medical society expelled one of their respectable members, for administering a remedy in *certain cases,* which *he found* to be beneficial? * * * * Can a member say, if *he* has found it more beneficial than any thing, in *certain cases,* that it is malpractice in those cases? Will you make a man swear to a lie? Will you stretch him on the bed of Procostes,* break him on the wheel, impale, or crucify him, because he cannot be made to chime in, exactly with a portion of a society? * * * * * I humbly trust, I know the powers reposing in our society, and the rights of its individual members, and I boldly assert, that our society have *no right* to coerce any member, in his professional practice. He is answerable to his God, and his patients—they will judge him—by his practice, he will rise, or fall." This correspondent closes his argument by saying, "At any rate I shall object to being turned out."

A sentiment similar to that expressed in the letters above quoted, is expressed by resolutions passed by the Virginia Society of Surgeon Dentists, at their third annual meeting, in October last; and particularly is this sentiment prominent, in the record of the transactions, accompanying these resolutions, in relation to which, it is observed, that there was "but one dissenting voice." The resolutions are as follows, viz. "*Resolved,* by the Virginia Society of Surgeon Dentists, that we believe the use of all PASTES and CEMENTS, of which MERCURY is a part, entirely unfit for, and highly objectionable, as fillings for carious teeth, that the use of them, in dental practice is empirical, AND IS HEREBY DECLARED TO BE MALPRACTICE.

"*Resolved,* That while we reprobate the use of all such mercurial preparations, and will execute our laws with fidelity and promptness, we claim no authority over the *opinions* of our members, nor will we ever require of

* Procrustes.

them, other pledges, than those which *exist* among honorable men, united for the purpose of improving and elevating a noble science."

The following record immediately follows the resolutions: "Some debate arose upon the passage of these resolutions, whether the Society had *power* to expel its members for their *opinions,* or to convert its executive officers into father confessors, to receive the confessions of delinquents, and grant absolution for offences. It was conceded by all that participated, that the society was not a court of *conscience,* and that *they had no right* to demand any expression of *opinions,* or to require any pledge from them in relation to the use of mercurial pastes and cements. * * * To expel for refusing to express an *opinion;* or to give a pledge, was making the *refusal* malpractice, which was *nonsensical and absurd.*" It was also contended, that to require action under a THREAT, was a course of procedure, to which no honorable man would submit.

It is unnecessary to multiply quotations, to get a clear exhibition of the objections, or rather the objection, of those above alluded to; it may be briefly stated as follows:

The American Society of Dental Surgeons had no *right* to adopt the measure in question.

Now, in respect to this question of RIGHT, we have yet to learn, that a voluntary association, untrammelled even by a *charter,* as is the American Society of Dental Surgeons, has not the *right* to pass *any* measure they please to the extent of expulsion of their own members. There cannot be the least shadow of doubt, that, so far as the *right* is concerned, they would have a most undoubted *right* to pass an ordinance declaring it malpractice to fill teeth with gold, or that the use of concentrated sulphuric acid as a *wash* for the teeth, was good practice; although either of these positions would evidently be most unreasonable. But we contend that their unreasonableness would not necessarily curtail the right, nor, on the other hand, the right make the request reasonable. They would not only have a *right* to pass such an ordinance, but they would have an equal right to expel a member, for non-compliance therewith. But this, it may be remarked, would be the extent of their power. They would have *no right* to declare the offence of transgression punishable with death, nor, indeed, to demand any expiation whatever, except a forfeiture of membership. Now, if our objectors mean, by saying that the American Society *had no right* to pass the offensive resolutions referred to, that these resolutions are unreasonable or unjust, this constitutes an entirely new issue—a ground which we might concede without in the least altering our position in respect to the right of the Society. So clear, in our mind, is it, that *any voluntary associations,* accountable to no authority save the laws of the land and sound morality, have a most perfect right to adopt their own rules of government, prescribe their own conditions for membership, and to expel for non-compliance, that we consider any attempt to sustain the position but a waste of words. But, as the American

Society has been charged with *usurpation* and *injustice*, not only by individuals, but by an organized and *chartered* association, whose constitution and by-laws are but a *transcript* of those adopted by the American Society, having, hence, the same professed objects, and governed by the same laws, we may well inquire into the *reasonableness* and *justice* of the resolutions originating this grave charge. As the resolutions and the accompanying record of the Virginia Society of Surgeon Dentists embody the precise objections made by the individuals whose letters are above quoted, we shall confine our remarks mainly to the transactions of this Society, or the record made of them. In pursuing this investigation, we may remark, that it seems to us that this honorable body have aimed their whole musketry at a *mistaken* object, or have set up an imaginary one, and are claiming credit for demolishing it. The very extraordinary assertion is made in one of their resolutions upon this subject, that *they "claim no authority* over the *opinions* of *their* members." Do they not require, nay, *compel* their members to *sign* their constitution, and to *adopt* their by-laws? And do not both their constitution and by-laws contain *opinions* and sentiments which are thus *forced* upon their members? Nothing is more clear. But they do not stop here. We find, on page 10, of their pamphlet, entitled "Transactions of the Virginia Society of Surgeon Dentists," under the head of RESOLUTIONS HAVING THE ACTION OF BY-LAWS, a resolution, declaring the *use* of all amalgams for dental purposes, as empirical and as malpractice!

Now, if this resolution is brought on a par with the by-laws of the Society, we ask if *their* members are not compelled to adopt *their* sentiment in connection with the by-laws, and to pledge themselves to sustain it. We say then, even on the supposition that the circular was intended to force an *opinion*, or to require a pledge, in respect to an *opinion*, and that non-compliance with this request was to be punished by expulsion, the American Society would have done no more or less than is in fact done by the Virginia Society, who have sought to cast reproach upon the measures adopted by the former. But we by no means admit this to be the import of the circular.

The *opinion* that the use of all amalgams is malpractical, had stood recorded not only upon the book of record, but in the Journal, the organ of the Society, for *four* years, at the time the circular was ordered. This opinion every man was as much bound to adopt as any precept in either the constitution or by-laws, and all *had*, by their silence, at least *tacitly*, adopted it. For regarding this *tacit* adoption as an adoption in *fact*, we have a precedent in the decision of this very Society, at their annual meeting, held in the city of Richmond, October 11, 1843, and published in the American Journal of Dental Science, vol. iv. page 115. The record is as follows:

"The committee appointed by the Virginia Society of Surgeon Dentists, to investigate certain charges alleged against Dr. R. N. Hudson, a member of said society, beg leave to report, that having examined the testimony upon which these charges were founded, and politely and respectfully offered him an *opportunity* to vindicate himself, which he most abruptly and rudely *declined, thereby,* in the opinion of the committee, TACITLY *acknowledging the truth* of the allegations," &c. "*Whereupon,* it was *Resolved,* that the name of Dr. R. N. Hudson be *stricken from the roll of members* FOR CONTUMACY!!!" This member was not, by this resolution of the VIRGINIA SOCIETY OF SURGEON DENTISTS, expelled for *malpractice,* but for *contumacy,* for contempt of the precepts and action of the society. For the benefit of those who may not be in possession of the full series of the Ameriean Journal of Dental Science, we will here give the several resolutions adopted by this society, upon the subject of mineral paste. The first was adopted at the second annual meeting, held in Philadelphia, August, 1841, and is as follows:

"The Committee, Drs. E. Parmly, E. BAKER, S. Brown, C. A. Harris and J. Parmly, to whom the duty of reporting on the use of lithodeon, mineral paste and all other substances of which mercury is an ingredient for stopping teeth, reported, in substance, that the use of all such articles, were hurtful, both to the teeth and every part of the mouth; and that there was no tooth, in which caries in it could be arrested, and the organ rendered serviceable by being filled, in which gold could not be employed. The report was *unanimously* adopted by the society." Now, what constitutes the American Society of Dental Surgeons, if not its members? The next was adopted at the fourth annual meeting, held in Baltimore, July, 1843, and is as follows:

"On motion of C. A. Harris, *Resolved,* that this society regard the use of mineral paste, in plugging carious teeth, as malpractice, and that a committee of three be appointed to receive information and facts, on that subject, to be transmitted to Dr. Westcott of Syracuse, in the state of New York, to be by him laid before the Medical Society of the county of Onondaga, in that state, before which the subject aforesaid is now pending." This resolution was also unanimously adopted.

The third resolution was adopted at the fifth annual meeting held in the city of New York, Aug. '44, and is as follows:

"*Resolved,* That the Recording Secretary be instructed to give notice to every member of this society, who is charged by another member, with plugging carious teeth with mineral paste, that this society have pronounced the use of this article, for this purpose, as malpractice; and that each member persisting in its use will have his case acted upon at the next annual meeting."

Now, who claiming to be a member of the American Society, will contend that he has not committed himself, in respect to his *opinion* upon the use of amalgams, after these resolutions have been for years a matter of record in the American Journal? If any were opposed in *opinion* to the views of the Society, why had they not made it known, and asked the society to excuse them from expressing an opinion in common, which they could not tolerate as private individuals? But if the circular was not intended to elicit *opinions*, the question very naturally arises, what was its intent? To give this clearly, it will be necessary to allude to some of the circumstances which constrained the society to adopt this measure, in order to effect a thorough renovation in respect to this practice.

By a reference to the resolutions of 1844, it will be seen that some *action* must be taken upon the subject at this session—hitherto all had been talk. Accordingly, the society appointed a *committee* of investigation. This committee after a most patient and thorough investigation, made a report containing details no less mortifying than astonishing. This report was in substance, that, of the twenty-five members resident in the cities of New York and Brooklyn, all were called upon, except two who were absent, and two who had ceased to practice. Of the remaining twenty-one, only ten disapproved *entirely* of the use of amalgams for dental purposes!!

Of the eleven remaining, five used the amalgams in certain cases, but were willing to pledge themselves to abandon it altogether.

Six used amalgams in certain cases, and *refused* to discontinue its use.

It appears from the above statistics, that nearly one-fourth of all the members resident in the cities of New York and Brooklyn not only used it, but openly disregarded the action of the Society upon the subject. In other words, one-quarter of the members in those cities were engaged in a kind of practice, which the society, by strong resolutions, had *thrice* declared to be malpractice.

Here we may properly inquire whether the society had a right to appoint this committee, and authorize them to make the investigation which elicited the above facts. Will not even our Virginia friends justify the society for creating such a committee—or would they have relied upon the "pledges which EXIST among *honorable men*, united for the purpose of improving and *elevating* a noble science." The American Society had been relying upon such pledges for years, but the facts elicited by this committee constrained them to feel that they had been imposed upon. Under these circumstances one of two conclusions must be adopted. The honorable members of the society must either submit to be disgraced, by fellowshiping members whom they had declared to be engaged in malpractice, thus resigning all hope of any distinction which membership could confer, and all influence which the society could under such circumstances command; or they must take some measures to rid themselves of these members. The latter was the conclusion adopted. But how was this to be effected? The society could not send their committee over the entire globe to make the necessary investigations. The *circular* was therefore proposed to supply the place of the committee. This could travel with more despatch and much cheaper, and could do the work with equal efficiency—the object of either being to reduce, to a certainty, the number of those who were willing to redeem the pledge, which every member had tacitly given, upon this subject, and to declare to each member, that unless he *did* redeem his clearly implied pledge, that he should no longer be considered worthy to be a member of the society. And here is the "*threat*" which the Virginia Society holds up as such a terrible monster! But if *this* be regarded in the light of a *threat*, the constitution itself, and every law, having a *penalty* attached, must be regarded in the same light. Such is *indeed* the case, in respect to all who have violated the law, but to no others.

We by no means say that *all* who refuse to sign the circular issued by the American Society, either have used amalgams for dental purposes, or that they are inclined to do so, yet we boldly affirm, that the "tone" of the circular "is *threatening*" to no others, and that it should prove obnoxious to any others, is to us a matter of the greatest surprise. We have thus far considered the offensive measure of the American Society as being simply a measure to *execute* their own laws, and one which they of course had a most perfect right to adopt. The society had *abundant* evidence, that *some* of her members, had been robbing her of her rights and her honors, and she accordingly issued this "*search warrant,*" that she might bring the rogues (only) to justice. We have endeavored to show that the measure was not intended to coerce an *opinion*—that it was not intended to elicit any *new* opinion, but simply to ascertain who had been true to themselves, and to the society, and who were willing to abide by her settled, and recorded maxims. We think that we do not misconstrue the spirit of the Virginia Society, in their resolution and remarks concerning the action of the American Society, when we say, that the former society clearly give to *their* members, the liberty to advocate any *opinion* they please—to preach as they please, if they will only *practice* right. In other words they may proclaim to *all*, that mineral paste, is the very best known substance with which to fill carious teeth, if they will not *use* it. But *using* it, would be malpractice.

But let us for a moment examine this sage doctrine. Possibly it may be shown to be bad policy, if not "nonsensical and absurd."

For *ourself*, we should consider it, neither *unjust*, "*nonsensical*," nor "absurd," to *expel* a member for *advocating* the *opinion* that mercurial pastes were fit articles for stopping teeth, certainly if he was a member of either the *American* or *Virginia* Society, with *their present recorded* precepts upon this subject. If it could not be regarded malpractice it would be a very mal-opinion, and one which would not only disgrace himself, but the society, and the profession. *Why* is a member expelled for malpractice in dentistry? Is it because he has, by such practice, injured a fellow-being? If so, does not the same charge lie against one, who by his *opinions*, encourages, and sustains; others, in malpractice, perhaps thereby, causing many more to suffer, than would have been the case, had *he himself*, been the malpractitioner? Or is one to be expelled from a society, for malpractice, because such practice tends to disgrace the society, and the profession? If so we ask how much less disgrace, does he bring upon either, by giving the *opinion*, that what the society have denounced in the strongest terms, as malpractice, is nevertheless good practice, and but for the "*bond*" he would adopt it?

Is it not true, that any position which would prevent one's obtaining membership, ought to deprive him of membership, in any voluntary association? Would not a candidate for admission into any particular church, be justly excluded, should he, on having the creed of said church presented to him for his adoption, take the ground he had a right to *his own* opinions, and that he was not bound to express an opinion? And would not a *member* be responsible to said church, should he at *any time*, and especially after he *had* adopted its *opinions*, be responsible for such a position? The author of our second letter, backed by the Virginia Society of Surgeon Dentists, would say no—by no means! Such person "would only be responsible to his God," and to excommunicate such a member, for *refusing to give an opinion*, would be "nonsensical and absurd." Now what would be the precise rights of each party in this case? In respeet to the candidate, he would have an undoubted right to *apply* to any church for admission, whatever opinion he might entertain of its doctrines. But if his views did not coincide with the established maxims of the church, to which he applies, he would have no right to expect, much less demand, admission. The church, on the other hand, would have a right to refuse him admission. On the supposition that he had already obtained membership, it would be equally clear that the church would be fully authorized to dismiss him for a similar position.

Now could this man make the plea, and be sustained, either by usage, common sense, or public opinion, that the church had undertaken to coerce an opinion? Precisely this, is the position of the American Society. Far be it from this society, to wish, to coerce opinion. She does not believe in "forcing men to volunteer." But for an institution framed almost for the sole purpose of suppressing quackery, *to be forced* to retain members, who *advocate* what she has decided to be the worst form of quackery, and who refuse even to give an opinion adverse to such practice, is a rare doctrine—one which we think may be considered both "nonsensical and absurd." To say the least, it is one which chimes but poorly with the spirit originating the American Society of Dental Surgeons. One of the most prominent objects of this association, as set forth in the preamble to the constitution, is, to correct abuses in dental practice or to suppress quackery. Now we ask, does a member of this association, as every member is bound to do, help to carry out this object by expressing an *opinion* that the use of mineral paste is good practice? Does he not rather take the most efficient way possible to thwart this object? And ought the *society to be compelled* to fellowship such a member, even though his offence is a mere matter of *opinion?* We may illustrate the pernicious *effect* of this morbid liberality, which constitutes the basis of such unrestrained freedom of *opinion,* by a case, connected with this very subject. While this subject was pending before the medical society of this county, Dr. Bliss, the chief champion on the side of mineral paste, sought to convince said society, and the public, that cement was *the substance* of all substances, for filling teeth, by collecting and exhibiting the *opinion* of every amalgamite, far and near. To this end he went to the city of New York, and there obtained, as the phrase goes, "a splendid collection." On his return, he took good care to have it well understood, that he had got the opinion of several of the members of the American Society of Dental Surgeons, that mineral paste might be judiciously used for filling teeth. Now, although these *opinions* did not prevail with the medical society, they undoubtedly did induce hundreds to have their teeth filled with this vile material. When this subject came up at the last annual meeting, one of those who had given his favorable opinion (in writing) of the practice, to Dr. Bliss, said he was willing to discontinue its *use,* but had a right to his *opinion.* It was contended by the society, that inasmuch as he had given currency to his opinion, that this article in question was good, and that, by a *certificate,* he was bound either to free them from the responsibility of his opinion, by leaving the society, or he was bound to undo his act (the expression of his opinion) in the same way, viz. by signing the proposed certificate, or protest of the society. But this he refuses to do, on the ground that the society have no right to make such a demand! As we have introduced this member, to exhibit the consistency of those who claim a seat in the society, while they disregard both its precepts, and its action, we will refer to his positions on several different occasions. We find him one of the foremost in carrying out measures to expel, not from the society, but from the *country,* Monsieur Mallallan, for *the* malpractice of using mineral paste. We next find him at Philadelphia, *one of the committee* which originated the resolution, there passed by the society, in 1841, declaring, mineral paste "hurtful both to the teeth, and every part of the mouth."

We next find him in New York, *filling teeth* with this very mineral paste!

We next hear of him through Dr. Bliss, as the author of one of his many certificates in *favor* of this practice; and lastly, we find him as the author of the second letter above quoted, denouncing the American Society of Dental Surgeons as having "transcended their powers." But in this last resort he has nothing to fear, being fully backed, and sustained in this position by the Virginia Society of Surgeon Dentists!

We have no doubt this honorable body will have occasion to extend a like sympathy to many others, as persecuted members of the American Society, as they do now extend the privilege to *their* members, not merely to advocate the use of amalgams, but to give *written* certificates in its favor.

In conclusion, we may say that this measure of the American Society of Dental Surgeons, adopted to *execute* her laws, will be sustained—not merely by a large majority of her members, but by common sense, and by public opinion. The certificates *already* received, duly signed, show that the society have nothing to fear from those who raise the cry of usurpation. The society have asked no member to *prove* himself innocent, but have offered to take his own statement, as sufficient proof of the fact. All admit that the society have full power to expel *on* conviction, and does not the refusal of a member to answer for himself, clearly imply his guilt? This, in the language of the Virginia Society, is at least a "*tacit* admission" of it, and this body have decided that a *tacit* admission, is an admission in fact, and have given us a *precedent,* for striking "from the roll of members" all guilty of such *contumacy.—Syracuse Ed.*

